# Knockdown of RNA Interference Pathway Genes in Western Corn Rootworms (*Diabrotica virgifera virgifera* Le Conte) Demonstrates a Possible Mechanism of Resistance to Lethal dsRNA

**DOI:** 10.1371/journal.pone.0157520

**Published:** 2016-06-16

**Authors:** Ana María Vélez, Chitvan Khajuria, Haichuan Wang, Kenneth E. Narva, Blair D. Siegfried

**Affiliations:** 1 Department of Entomology, 103 Entomology Hall, University of Nebraska-Lincoln, Lincoln, Nebraska 68583–0816, United States of America; 2 Dow AgroSciences, 9330 Zionsville Road, Indianapolis, Indiana 46268, United States of America; 3 University of Florida, Department of Entomology and Nematology, PO Box 110260, Gainesville, Florida 32611, United States of America; Institute of Plant Physiology and Ecology, CHINA

## Abstract

RNA interference (RNAi) is being developed as a potential tool for insect pest management. Increased understanding of the RNAi pathway in target insect pests will provide information to use this technology effectively and to inform decisions related to resistant management strategies for RNAi based traits. Dicer 2 (Dcr2), an endonuclease responsible for formation of small interfering RNA’s and Argonaute 2 (Ago2), an essential catalytic component of the RNA-induced silencing complex (RISC) have both been associated with the RNAi pathway in a number of different insect species including the western corn rootworm, *Diabrotica virgifera virgifera* (Coleoptera: Chrysomelidae). We identified both genes from a transcriptome library generated from different tissues and developmental stages of the western corn rootworm, an important target pest for transgenic plants expressing dsRNA targeting essential genes. The expression of these genes was suppressed by more than 90% after injecting gene specific dsRNA into adult rootworms. The injected beetles were then fed *vATPase A* dsRNA which has previously been demonstrated to cause mortality in western corn rootworm adults. The suppression of both RNAi pathway genes resulted in reduced mortality after subsequent exposure to lethal concentrations of *vATPase A* dsRNA as well as increased *vATPase A* expression relative to control treatments. Injections with dsRNA for a non-lethal target sequence (*Laccase 2*) did not affect mortality or expression caused by *vATPase A* dsRNA indicating that the results observed with *Argo* and *Dicer* dsRNA were not caused by simple competition among different dsRNA’s. These results confirm that both genes play an important role in the RNAi pathway for western corn rootworms and indicate that selection pressures that potentially affect the expression of these genes may provide a basis for future studies to understand potential mechanisms of resistance.

## Introduction

RNA interference (RNAi) refers to the suppression of gene expression by small noncoding RNA molecules, and was first reported by Fire et al., [1] who demonstrated that application of exogenous dsRNA can be used to suppress the expression of the homologous messenger RNA (mRNA) thus rendering the gene to be non-functional. The RNAi process requires a complex set of proteins working together and involves several steps. In brief, when exogenous dsRNA is introduced into the cell, it is processed by the ribonuclease III enzyme, Dicer 2 (Dcr2), into small ~21 nucleotide sequences, referred to as siRNAs [2]. These siRNAs are then picked up by the RNA-induced silencing complex (RISC) and are unwound to become a single strand that is referred to as the guide strand. The RISC complex along with the guide strand pairs with the homologous mRNA which is then cleaved by the RISC-bound Argonaute 2 protein [3]. Argonaute 2 (Ago2) protein contains two distinctive domains: a PAZ domain and a PIWI domain [4]. The PAZ domain has been suggested to be involved in the RNA binding whereas the PIWI domain is similar to RNase H in structure and function, and causes the cleavage of the target mRNA [3].

Although RNAi has been widely described with variable success in at least 30 insect species to determine gene function [5], relatively few studies have been conducted in insects to identify the key components of the RNAi pathway. *Dicer 2* (*Dcr2*) and *Argonaute* 2 (*Ago2)* sequences have been identified in many insects including the model species *Tribolium castaneum* (Herbst) and *Drosophila melanogaster* Meigen. In *T*. *castaneum*, *Dcr2* and *Ago2* have been reported to be involved in the RNAi pathway as suppression of these genes by RNAi reduced the efficiency of RNAi mediated knockdown of enhanced green fluorescence protein (EGFP) in transgenic beetles (enhancer trap line, *pu-11*) that have been engineered to express *GFP* [6]. In addition, Lee et al. [2] identified *D*. *melanogaster* mutants with base pair substitutions of the *Dcr2* gene that significantly altered the predicted protein product and observed that these mutants were defective for processing siRNA precursors. Similarly, Okamura et al. [7] produced a *Drosophila* strain bearing deletions in *Ago2* and thus lacked both *Ago2* mRNA and protein. These authors observed that eggs laid by these mutants were defective in the RNAi response as eggs injected with dsRNA for *fushi tarazu* (*ftz*), a *Drosophila* segmentation gene, did not produce the phenotype for flies lacking *ftz* expression.

A number of recent studies have demonstrated that transgenic plants expressing insect-specific dsRNA can be effectively used to manage insect pest species [8–10]. One of the targets for this technology is the western corn rootworm (WCR), *Diabrotica virgifera virgifera* LeConte. This species is a particular challenge to manage because of its sequential ability to evolve resistance to insecticides including transgenic corn plants expressing *Bacillus thuringiensis* toxins [11–14]. Recently, Baum et al. [10] screened several hundred potential target genes for RNAi knockdown and subsequent mortality by allowing the WCR larvae to feed on artificial diet treated with dsRNA. In the same study, the authors demonstrated that transgenic corn plants expressing dsRNA for subunit A of the housekeeping gene vacuolar *ATPase subunit A* (*vATPase A*) can effectively protect roots from rootworm feeding and thus established RNAi as a potential tool for rootworm management [10].

To evaluate the potential of RNAi as a pest management technique, it is critical to understand the key components of the RNAi pathway in general and specifically in WCR. Miyata et al. [15] recently demonstrated that silencing of *Ago2* and *Dcr2* in western corn rootworms resulted in antagonism of an RNAi mediated silencing of critical pigmentation/tanning genes using an *in vivo* assay system. However, the phenotypic responses associated this assay system were only tested in larvae and effects of *Ago2* and *Dcr2* knockdown on lethal RNAi responses have not been examined. In the present study, we describe a combination of bioassay and gene expression studies involving RNAi mediated knockdown of both a lethal target (*vATPase A*), a non-lethal rootworm target sequence, *laccase 2* (*lac2*), and two putative pathway genes, *Ago2* and *Dcr2*. Results of this investigation demonstrate the involvement of these pathway genes in the RNAi response of WCR adults. Importantly, because competition among multiple RNAi treatments may cause reduced efficiency of individual responses [6, 15, 16], we also show that such competition is not responsible for the antagonism of toxicity or knockdown associated with either *Dcr2* or *Ago2*. These results provide a basis for future studies aimed at improving RNAi for WCR control and for investigation of potential resistance mechanisms for lethal RNAi target sequences.

## Methods

### Identification of genes involved in RNAi pathway

Transcriptome sequencing of *D*. *v*. *virgifera* has been previously described [17]. Using Illumina paired-end as well as 454 Titanium sequencing technologies, ~700 gigabases (700 billion bases) were sequenced from cDNA prepared from eggs (15,162,017 Illumina paired-end reads after filtering), neonates (721,697,288 Illumina paired-end reads after filtering), and midguts of third instars (44,852,488 Illumina paired-end reads after filtering). *De novo* transcriptome assembly was performed using Trinity [18] for each of the three samples as well as for the pooled dataset and the pooled assembly resulted in 163,871 contigs (the average length: 914 bp). To identify the sequences from WCR associated with the RNAi pathway, we downloaded from GenBank, the *T*. *castaneum* sequences encoding for genes with a putative role in the RNAi pathway and blasted them locally against our WCR transcriptome database.

### Double stranded RNA (dsRNA) preparation

Total RNA was isolated from the whole bodies of WCR adults using RNAeasy mini Kit (Qiagen, Valencia, CA) following the manufacturer’s recommendations. Total RNA (1 μg) was used to synthesize first strand cDNA using the Quantitech Reverse Transcription kit (Qiagen, Valencia, CA). Primers were designed using Beacon designer software (Premier Biosoft International, Palo Alto, CA) and T_7_ polymerase promoter sequences were placed in front of both forward and reverse primers (Table 1). *D*. *v*. *virgifera laccase2* was used as non-lethal target gene since it represents a non-lethal gene that encodes a phenoloxidase required for cuticle sclerotization and pigmentation in larvae but should not affect mature adults [19]. The same primers reported by Rangasamy and Siegfried [20] were used for the amplification of *vATPase A*. For a negative control, a non-specific *GFP* (green fluorescence protein) gene was amplified from the pIZT/V5-His expression vector (Invitrogen) using the gene-specific primers given in Table 1. The PCR product amplified for WCR RNAi pathway genes and *GFP* were used as a template for *in vitro* synthesis of dsRNA’s using the MEGAscript transcription kit (Applied Biosystems Inc., Foster City, CA). All the synthesized dsRNA’s were purified using the RNAeasy Mini kit (Qiagen, Valencia, CA) following the manufacturer’s recommendations. All dsRNA preparations were quantified using a Nanodrop spectrophotometer (Thermo Scientific, Franklin, MA) and analyzed by electrophoresis to determine purity.

**Table 1:** Sequences and product length for primers used in synthesis of dsRNA.

Gene Name	Primer sequences	Product Length
*Dicer 2*	F: TAATACGACTCACTATAGGGATACAGGTTCAACAACAAGG	377
	R: TAATACGACTCACTATAGGGCTGCCAGTTGGTATTCATC	
Argonaute 2	F: TAATACGACTCACTATAGGGATCTCTTGGATTCAATGGGA	366
	R: TAATACGACTCACTATAGGGCCTGATTCGCAACATATACC	
vATPaseA	F: TAATACGACTCACTATAGGGTATTGTACAGGTG	258
	R: TAATACGACTCACTATAGGGCAATTTCCAAG	
*Laccase 2*	F TAATACGACTCACTATAGGGATGTGCAAGAGCTTGTAGGG	183
	R: TAATACGACTCACTATAGGGATGCGATTGGCTGTTAGAAG	
*GFP*	F: TAATACGACTCACTATAGGGGGTGATGCTACATACGGAAAG	370
	R: TAATACGACTCACTATAGGGTTGTTTGTCTGCCGTGAT	

### Insect Bioassay

Non-diapausing WCR adults were purchased from the Crop Characteristics Inc. (Farmington, MN). All bioassays were conducted with the newly emerged beetles (within 48 hrs). Artificial diet was prepared using methods modified from Rangasamy and Siegfried [20]. Briefly, the diet consisted of 10% Invite EC (Florida Food Products, Eustis, FL) diluted in 4% agar, 0.32% methyl paraben, 0.12% sorbic acid, 0.16% streptomycin, to minimize fungal and bacterial contamination, and 16.6% honey. Immediately after obtaining adult rootworms, they were allowed to feed on the untreated artificial diet for 24 hrs before initiating the bioassays. To determine if dsRNA for *Ago2*, *Dcr2* and *lac2* caused mortality in rootworm adults, ~0.6 μl of dsRNA (1 μg/μl) was injected into each beetle using a glass needle and modified syringe. As a control, the same amount of *GFP* dsRNA or water was injected into the beetles. Before injections, insects were anaesthetized with carbon dioxide. Beetles without injections were also used as a control to estimate mortality associated with injection trauma. After injection, beetles were provided artificial diet every other day and reared for up to 18 days at 23±1°C and 75±5% relative humidity. Two cohorts of beetles with different dates of emergence were tested. Each cohort included three replications per treatment with 10 beetles for a total of 60 beetles per treatment.

**Fig 1:**
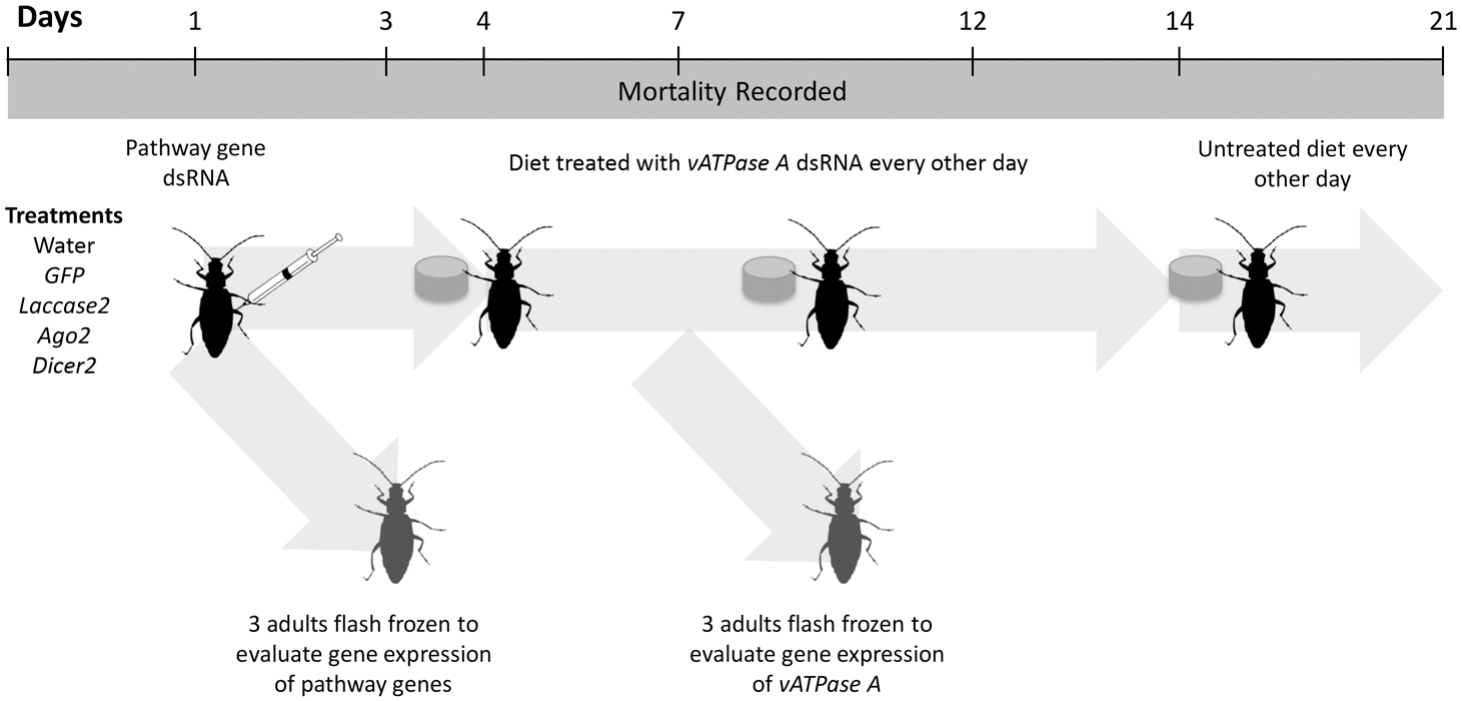
Two-step bioassay to identify the effect of the suppression of the pathway genes on WCR adults and to determine if the mortality caused by *vATPase A* dsRNA can be rescued by suppressing the expression of pathway genes.

To determine if the lethal effect of the *vATPase A* dsRNA can be antagonized by the knockdown of pathway gene expression, we utilized the design described in Fig 1. Adult rootworms were injected with ~0.6 μl of dsRNA (1 μg/μl) on day one and then allowed to feed on the untreated artificial diet. On day 4, beetles were transferred to a new container containing diet plugs (4 mm diameter×2 mm height) surface coated with *vATPase A* dsRNA (500 ng/diet pellet). New diet plugs treated with *vATPase A* dsRNA were provided every other day until day 12. For the remainder of the assay beetles were provided with untreated diet. Mortality was recorded every other day. On day 7, three beetles per replication were collected from all the treatments, flash frozen in liquid nitrogen, and stored at -80°C until total RNA was isolated for expression analysis.

### Real-time PCR

Total RNA was isolated from the whole bodies of adults using RNAeasy mini Kit (Qiagen, Valencia, CA) following the manufacturer’s recommendations. Before the initiation of the transcription reaction, the total RNA was treated with DNAase to remove any genomic DNA using Quantitech reverse transcription kit (Qiagen, Valencia, CA). Total RNA (500 ng) was used to synthesize first strand cDNA as a template for quantitative real-time quantitative PCR (qRT-PCR). Primers used for qRT-PCR analysis were designed using Beacon designer software (Premier Biosoft International, Palo Alto, CA) and are provided in Table 2. The efficiencies of primer pairs were evaluated using 5 fold serial dilutions (1: 1/5: 1/25:1/125: 1/625) in triplicate. Amplification efficiencies were higher than 96.1% for all the qRT-PCR primer pairs used in this study. All primer combinations used in this study showed a linear correlation between the amount of cDNA template and the amount of PCR product. All correlation coefficients were larger than 0.99 except for *Dcr2* which was 0.945 (Table 2). The 7500 Fast System SDS v2.0.6 Software (Applied Biosystems) was used to determine the slope, correlation coefficients, and efficiencies (Table 2). Three beetles from each replication, each with two technical replications were used for qRT-PCR analysis performed using SYBR green kit (Applied Biosystems Inc., Foster City, CA) and the 7500 Fast System real-time PCR detection system (Applied Biosystems Inc., Foster City, CA). qRT-PCR cycling parameters included 40 cycles each consisting of 95°C for 3 sec, 58°C for 30 sec, as described in the supplier’s protocol (Applied Biosystems Inc., Foster City, CA). At the end of each PCR reaction, a melt curve was generated to confirm single peak and rule out the possibility of primer-dimer and non-specific product formation. Relative quantification of the transcripts were calculated using the comparative 2-ddCT method [21] and were normalized to *β*-actin [20].

**Table 2:** Sequences and other relevant parameters for each primer pair used for expression analysis by qRT-PCR.

Gene Name	Primer Sequence	Product Length (bp)	Slope	R2	Primer Efficiency (%)
*Dicer 2*	F: TCGATAACTTCATGGCCCAAA	81	-3.20	0.948	105.0
	R: CCAGTTGGTATTCATCCTCCT				
*Argonaut*	F: AGCCCTGATTCGCAACATAT	109	-3.28	0.977	101.7
	R: TCTCCTGTCTGGGTGGTT				
*β-actin*	F: TCCAGGCTGTACTCTCCTTG	134	-3.42	0.99	96.1
	R: CAAGTCCAAACGAAGGATTG				
*v-ATPaseA*	F: GGAAGAAGATGATCTAGCCGAAATT	67	-3.36	0.993	98.4
	R: TTGTCCGTTTCTGCCAGAGA				
*Laccase 2*	F: GAGCAGCTTGCCAAGTATGT	109	-3.17	0.998	106.8
	R: TTGTCCGTTTCTGCCAGAGA				

### Statistical analysis

Gene expression and mortality were analyzed using SAS software version 9.3 [22] to determine differences between treatments. An analysis of variance (ANOVA) was performed using the PROC GLIMMIX statement with the least square estimated means procedure to determine differences between treatments.

## Results

### Identification of genes involved in RNAi pathway

To identify sequences of *Ago2* and *Dcr2* from WCR, we downloaded the *T*. *castaneum* sequences for both genes from GenBank and blasted them locally against a previously described WCR transcriptome database [17]. Our search identified sequences for *Dcr2* and *Ago2* with relatively high similarity (50–73%) to the *T*. *castaneum* sequences (Table 3). To determine if the WCR sequences have higher similarity with sequences from other organisms, these sequences were searched against the NCBI database using the Blastx search algorithm, and all sequences exhibited highest similarity to sequences from *T*. *castaneum*.

**Table 3:** Sequence identity and their best sequence match from Blastx search of the NCBI database.

Gene Name	Length (bp)	Organism	Accession #	Similarity (%)	E-Value	Score (bits)
*Dicer 2*	5044	*Tribolium castaneum*	NP_001107840.1	50	0.0	1624
*Ago 2*	3227	*Tribolium castaneum*	NP_001107842.1	59	0.0	959
*Lac 2*	504	*Tribolium castaneum*	NM_001039398.2	90	1E-106	332

**Fig 2:**
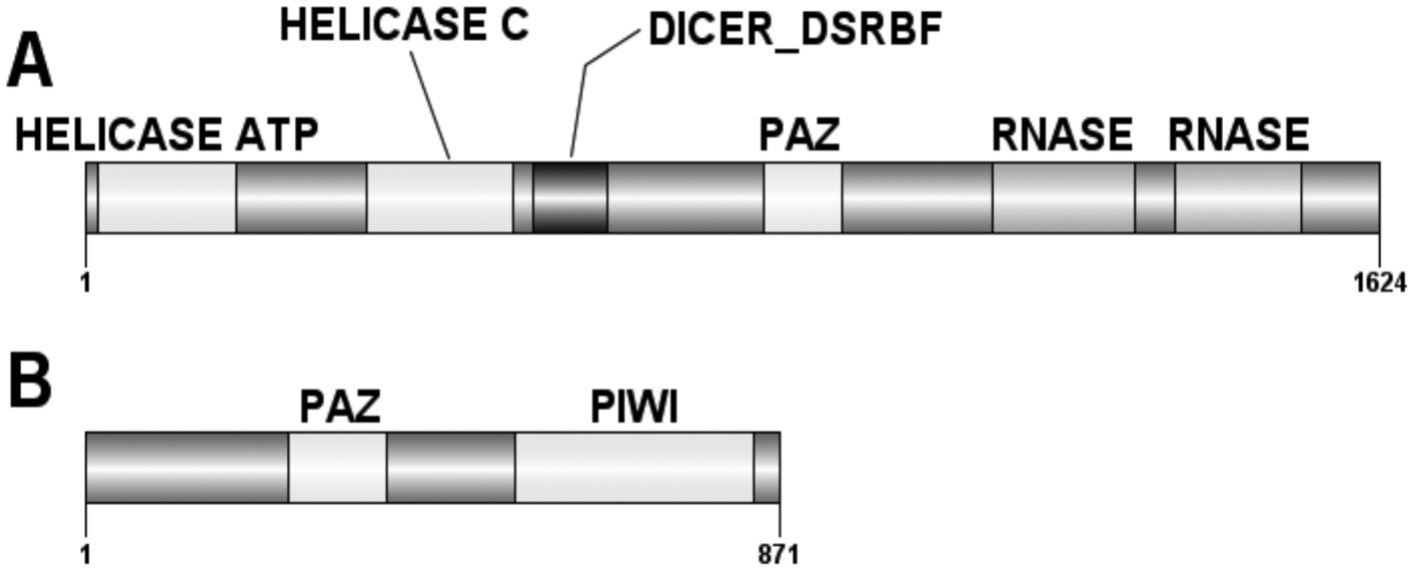
Domain analysis of the WCR RNAi pathway genes. (A) WCR DCR2 contain total of 6 domains, helicase ATP binding type 1 domain (helicase ATP), helicase C-terminal domain (helicase C), dicer double stranded RNA binding domain (dicer_dsrbf), PAZ domain and two ribonuclease III family domains (Rnase). (B) WCR AGO2 contains 2 domains, PAZ and PIWI. Domain’s for DCR2 and AGO2 were predicted using ScanProsite (http://prosite.expasy.org/scanprosite/).

Further analysis of these sequences indicated that both *Dcr2* and *Ago2* coded for full-length genes with 5,044 and 3,227 bp open reading frames (ORF’s), respectively. The translated WCR *Dcr2* is 1,624 amino acids in length and has two helicase domains at 15–191 and 353–536, a dsRNA binding domain at 561–654, a PAZ domain at 852–950 and two ribonuclease III family domains at 1,137–1,316 and 1,368–1,526 (Fig 2A). WCR *Ago2* is 871 amino acids in length and has two domains, PAZ at 256–378 and PIWI at 539–840 (Fig 2B).

### Suppression of RNAi pathway genes

**Fig 3:**
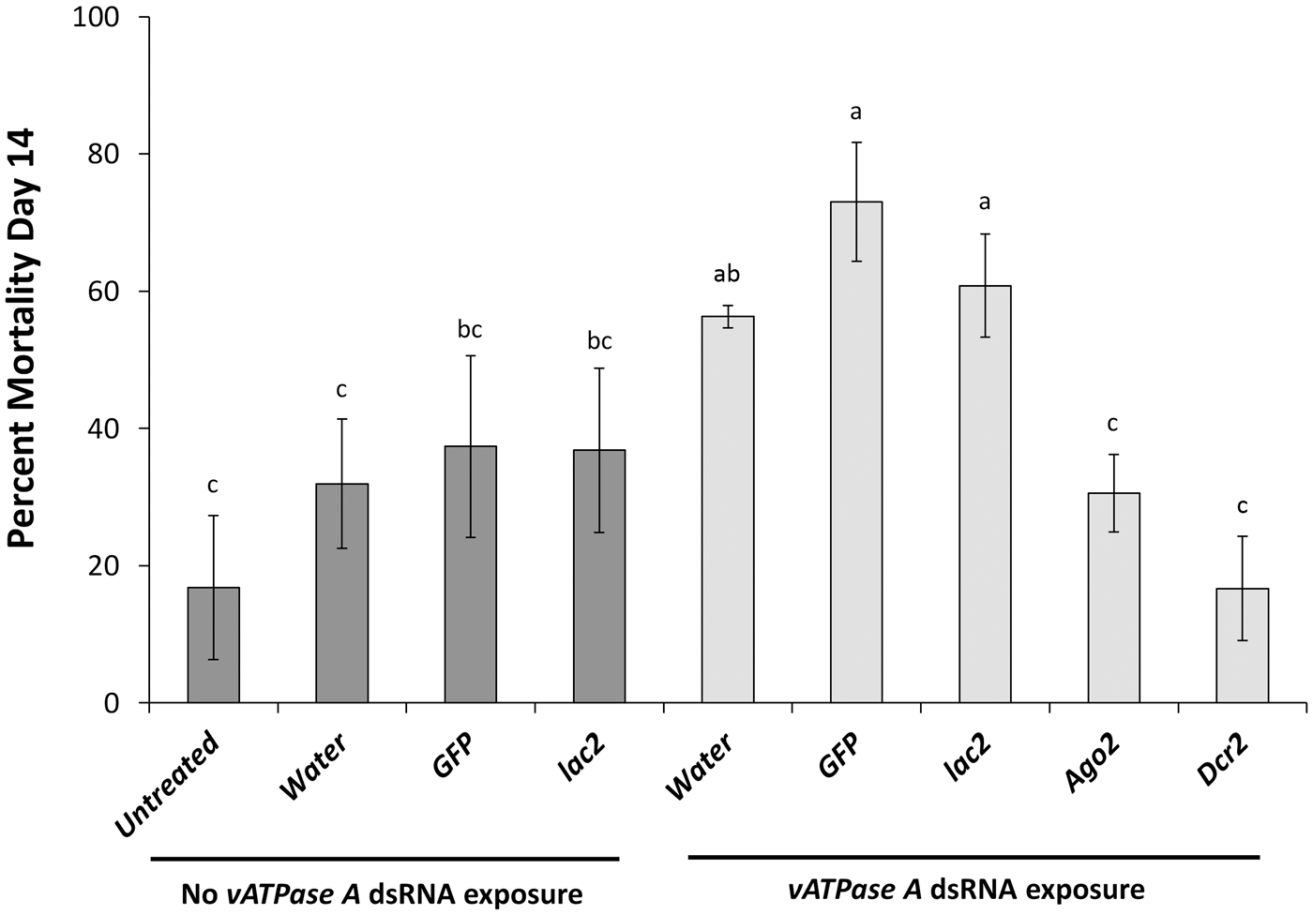
RNAi mortality suppression at day 14. Newly emerged beetles were injected with RNAi pathway gene dsRNA on day one. On day 4 adults were exposed to *vATPase A* dsRNA. Mortality was collected daily. Percent mortality at day 14 represents mean adult mortality calculated from two replications each containing 30 adults for a total of 60 beetles per treatment. Different letters represent significant differences at *P* value < 0.05.

To determine the effect of the reduced expression of pathway genes on the development of WCR adults, we injected beetles with *Dcr2*, *Ago2* and *lac2* dsRNA. There was no significant effect of these dsRNA’s at the concentration tested on the mortality of the beetles when compared with controls (beetles injected with water or green fluorescent protein (*GFP*) dsRNA). The mortality of injected beetles was also not significantly different from uninjected beetles maintained under identical conditions (Fig 3).

**Fig 4:**
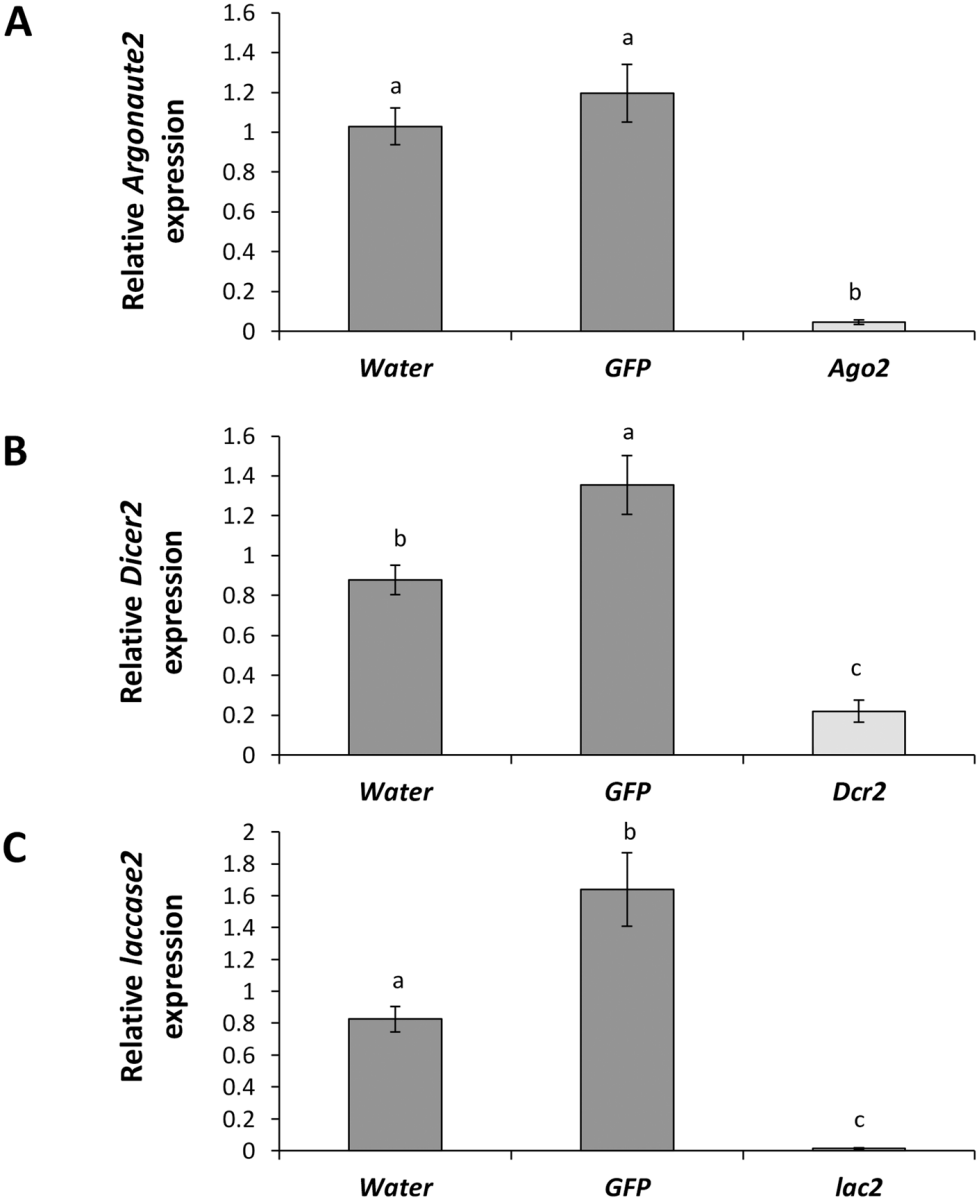
Relative expression of RNAi pathway genes and *laccasse2* in WCR adults. Newly emerged WCR adults were injected with 600 ng of *Argonaute2*, *Dicer2*, *laccase2* and *GFP* dsRNA or equal volume of water on day one and allowed to feed on untreated artificial diet. On day 4 adults were exposed to *vATPase A* dsRNA as surface treatment on artificial diet. On day 7 adults were collected and flash frozen in liquid nitrogen, and were used for gene expression analyses using qRT-PCR. (A) Relative *Argonaute2* expression. (B) Relative *Dicer2* expression. (C) Relative *laccase2* expression. Standard errors were determined from two biological replications, each including three individual beetles (n = 6), each with two technical replications. Different letters within a figure represent significant differences at *P* value < 0.05.

We also evaluated the expression of RNAi pathway genes and *lac2* in the beetles injected with specific dsRNA’s 7 days after injection (Fig 4). We observed over 96% reduction in *Ago2* expression relative to the water-injected beetles (Fig 4A). In *Dcr2* dsRNA injected beetles, we observed approximately 95% reduction in the transcription levels of *Dcr2* compared to beetles injected with water (Fig 4B). Similarly, beetles injected with *lac2* dsRNA exhibited over 98% reduction in *lac2* expression relative to water (Fig 4C).

### Suppression of RNAi-induced mortality

We have previously shown that feeding WCR beetles with *vATPase A* dsRNA causes ~100% mortality within 14 days of exposure to dsRNA [20]. To determine the role of pathway genes in RNAi induced mortality, we allowed beetles previously injected with water, *GFP*, *Dcr2*, *Ago2*, and *lac2* dsRNA to feed on artificial diet surface treated with *vATPase A* dsRNA at a concentration previously shown to induce mortality (500 ng/diet pellet) [20]. The mortality of beetles injected with water, *GFP* dsRNA or *lac2* dsRNA was not significantly different from mortality of untreated beetles. In contrast, beetles injected with water, *GFP* dsRNA or *lac2* dsRNA and subsequently fed with *vATPase A* dsRNA, exhibited significantly increased mortality relative to controls that were not fed with *vATPase A* dsRNA. However, the mortality of beetles injected with *Ago2* and *Dcr2* dsRNA was not significantly different from the untreated control (Fig 3) and therefore were apparently unaffected by the *vATPase A* dsRNA.

The mortality caused by *vATPase A* dsRNA was significantly reduced (P<0.05) at day 14 in the beetles with suppressed pathway genes compared to the beetles injected with water or *GFP* dsRNA and indicates their involvement in the RNAi response. Importantly, the mortality of beetles injected with *lac2* dsRNA and then fed *vATPase A* dsRNA was not significantly different from mortality observed in beetles injected with *GFP* dsRNA and water (Fig 3). Therefore, the reduced mortality observed in the *Ago2* and *Dcr2* treatments was not due to two different dsRNA’s competing for uptake and activation since the *lac2* dsRNA did not antagonize *vATPase A* toxicity. These results indicate that both *Ago2* and *Dcr2* are essential components of the RNAi pathway and to the lethal response associated with exposure to *vATPase A* dsRNA.

**Fig 5:**
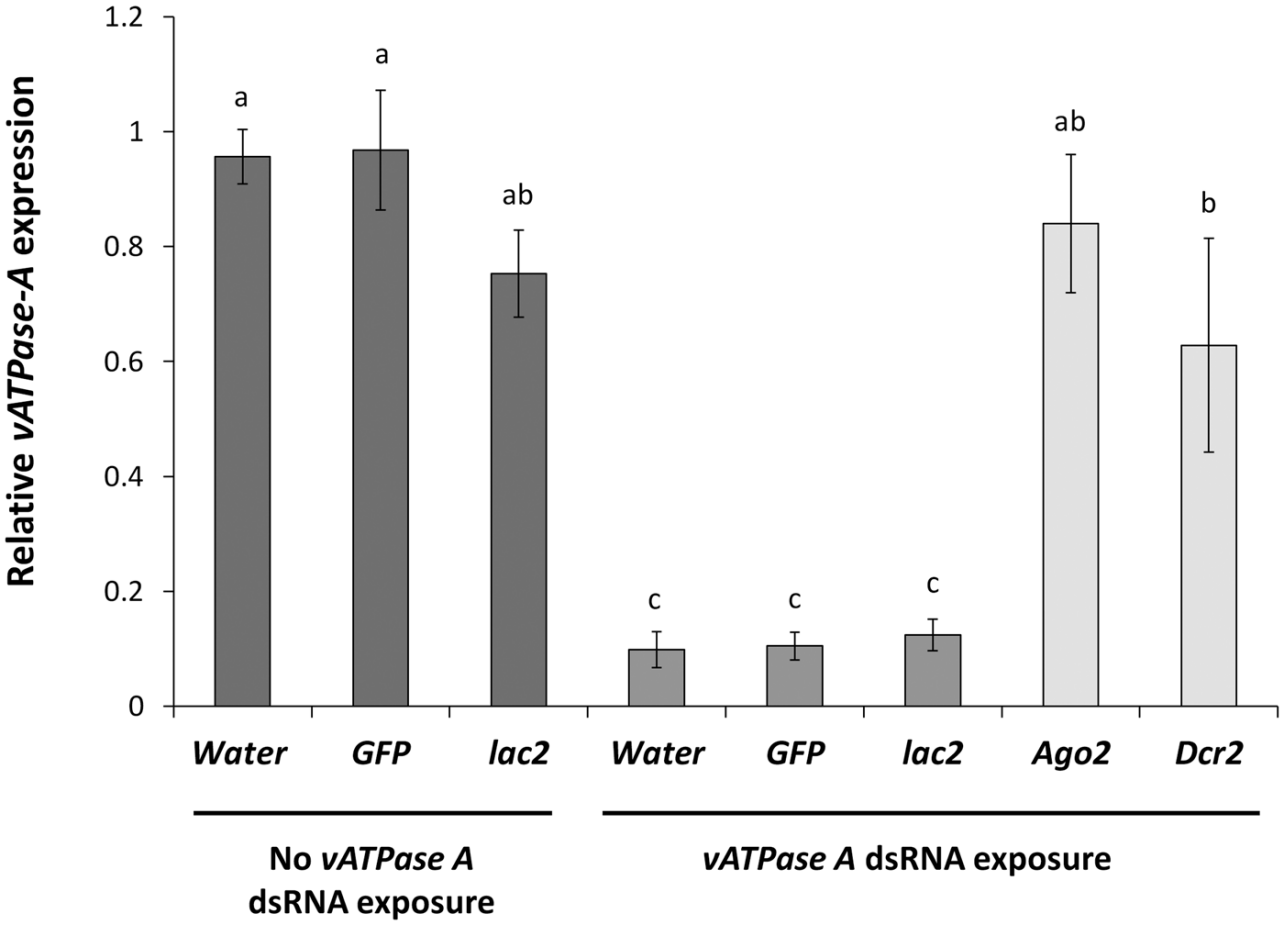
Expression analysis of *vATPase A* gene after both RNAi pathway gene dsRNA and *vATPase A* dsRNA exposures. Newly emerged WCR adults were injected with 600 ng of dsRNA or equal volume of water and allowed to feed on untreated artificial diet. On day 4 adults were exposed to *vATPase A* dsRNA as surface treatment on artificial diet. Three days after *vATPase A* dsRNA exposure, adults were collected and flash frozen in liquid nitrogen and used for *vATPase* expression evaluation using qRT-PCR. Adults that were only injected with water, *GFP* dsRNA and *laccase2* dsRNA but not exposed to *vATPase A* dsRNA were included as controls. Standard errors were determined from six independent biological replications (n = 6), each with two technical replications. Different letters within a figure represent significant difference at *P* value < 0.05.

We also evaluated the expression of the *vATPase A* in beetles which were first injected with *Ago2*, *Dcr2*, *lac2*, *GFP* dsRNA and then exposed to *vATPase A* dsRNA (Fig 5). Our results revealed that there was a significant reduction in the RNAi mediated suppression of the *vATPase A* gene in the beetles injected with *Ago2* and *Dcr2* dsRNA relative to expression in the beetles which were injected with water, *lac2* or *GFP* dsRNA and allowed to feed on *vATPase A* dsRNA (Fig 5). The percent suppression of the *vATPase A* expression in the beetles injected with dsRNA for the pathway genes was 15–37% compared to the controls which had 87–90% suppression (Fig 5). The expression of *vATPase A* in the beetles injected with *Ago2* and *Dcr2* dsRNA and fed *vATPase A* dsRNA was not significantly different from the expression observed in insects injected with water, *GFP* and *lac2* dsRNA and fed untreated diet (Fig 5).

## Discussion

RNA interference (RNAi) is a process that causes post-transcriptional gene knockdown in a sequence-specific manner and is initiated by introduction of dsRNA that is homologous to the silenced gene [23–26]. The potential to use RNAi as a pest management tool has been recognized for some time, and the recent demonstration that *in planta* RNAi can be used successfully for controlling western corn rootworm [10] has created an interest in understanding the RNAi pathway in this target pest. In this report, we have confirmed that *Dcr2* and *Ago2* play a critical role in the RNAi mechanism in WCR and importantly, that suppression of these RNAi pathway genes antagonizes the RNAi induced mortality caused by lethal target sequences of dsRNA in this species.

The importance of *Dcr2* and *Ago2* as components of the RNAi machinery has been documented in a number of insects. DCR2, a ribonuclease III enzyme, cleaves the dsRNA to produce many small ~21 bp siRNA’s [27], and AGO2 is part of the RISC complex that causes the cleavage of the target mRNA [3]. Our results indicate that the expression of WCR *Dcr2* and *Ago2* is reduced significantly by injecting the insects with the corresponding dsRNA’s and suppression of these genes is antagonistic to the mortality caused by subsequent feeding of *vATPase A* dsRNA. After 19 days the mortality of beetles injected with *Ago2* and *Dcr2* dsRNA and subsequently fed with *vATPase A* dsRNA was similar to untreated beetles (~40%). Furthermore, there was no significant effect on the survival or longevity of the beetles when pathway genes were suppressed by RNAi. A recent study with the brown planthopper, *Nilaparvata lugens* (Stål 1854) has also shown that reduced expression of the *Dcr2* gene did not affect the development and survival of insects exposed to dsRNA in artificial diet [28]. It is unclear how suppression of these genes might affect long-term fitness and survival in rootworms, but under laboratory conditions, there does not appear to be a significant impact from suppressed expression of these genes.

The results of this study strongly support that both *Ago2* and *Dcr2* identified in WCR, are critical to the RNAi pathway based on the reduced susceptibility to a lethal RNAi target sequence (*vATPase A*) after RNAi-mediated knockdown of these pathway genes. It should be noted that these results could be explained by competitive inhibition [6, 15, 16] that potentially results when dsRNA for two different target sequences (e.g., pathway genes and *vATPase A*) are co-administered. Such inhibition could explain the attenuated response both in expression and in mortality associated with exposure to the *vATPase A* dsRNA. Such competition has been reported previously [6, 15, 16] where a mixture of dsRNA can cause a saturation of the RNAi machinery and an inability to knockdown multiple genes at the same time. However, such competition seems unlikely, given that *lac2* dsRNA, which does not cause mortality in adult rootworms, did not cause a similar reduction in *vATPase A* expression or reduced mortality when beetles were fed *vATPase A* dsRNA. Moreover, to our knowledge, the evidence of competitive inhibition has always involved simultaneous exposure to multiple dsRNAs by injection. In our experiments the dsRNA for pathway genes were administered 48 hr prior to the second administration of the target sequence dsRNA (*vATPase A*) and therefore competition seems less likely.

The results of this investigation are consistent with those described by Miyata et al. [15] who also showed that RNAi of *Ago2* and *Dcr2* in western corn rootworm larvae reduced subsequent RNAi responses. Two genes (*laccase2* and *ebony*) that control larval cuticular tanning were chosen and the phenotypic response to silencing of these genes was visualized by changes in pigmentation during larval development. The RNAi response to *laccase2* and *ebony* dsRNAs by feeding was antagonized by prior feeding dsRNA for both *Ago2* and *Dcr2*. In this report, exposure to dsRNA for *Ago2* and *Dcr2* was achieved by feeding on artificial diet resulting in only moderate knockdown of both genes. In the system described in the present investigation, we were able to more accurately quantify the effect of RNAi of the two pathway genes using a lethal RNAi response, and by injecting the beetles with dsRNA for *Ago2* and *Dcr2*, we were able to achieve a higher percent knockdown for both genes. In addition, because adults are easier to maintain on artificial diet than larvae [20], this system may provide advantages over the larval assay system to assess the role of pathway genes in the RNAi response.

In conclusion, our results confirm the role of *Dcr2* and *Ago2* identified from WCR in eliciting an RNAi response in WCR adults. With the widespread use Bt crops and potential for WCR to develop resistance to a variety of insect management tools, there is a critical need for appropriate alternatives to transgenic Bt plants. One such alternative involves *in planta* use of RNAi. However, our results suggest that the RNAi pathway may represent a potential mechanism of resistance to lethal dsRNA’s, because the knockdown of these genes resulted in antagonism of the lethal response. A mechanism involving reduced efficiency of the RNAi pathway could provide broad resistance across a diversity of possible target sequences. Given the propensity for rootworms to evolve resistance to other management strategies, proactive identification of possible resistance mechanisms will enhance our ability to use the technology in a sustainable manner. Our results provide a basis for future studies aimed at improving RNAi technologies as a WCR management option.

## Supporting Information

S1 File Data supporting the results of qRT-PCR presented in Figs 4 and 5. (XLSX)

S2 File Data supporting the analysis of mortality after exposure to combinations of dsRNA presented in Fig 3. (XLSX)
